# Metastasising Pleomorphic Adenoma of the Parotid Gland: Where are We Now? A Systematic Literature Review

**DOI:** 10.1007/s12663-025-02472-w

**Published:** 2025-02-13

**Authors:** Sacchetto Andrea, Liberale Carlotta, Silvestrini Marina, Riva Giulio, Saetti Roberto

**Affiliations:** 1 Department of Otorhinolaryngology, San Bortolo Hospital, Viale Ferdinando Rodolfi, 37, 36100 Vicenza, VI Italy; 2 Member of Young Confederation of European ORL-HNS, Y-CEORL-HNS, Gothenburg, Sweden; 3 Unit of Otorhinolaryngology, Head & Neck Department Policlinico G. B. Rossi, University of Verona, Piazzale L.A. Scuro, 10, 37134 Verona, VR Italy; 4 Pathology Unit, Department of Diagnostics, San Bortolo Hospital, Viale Ferdinando Rodolfi, 37, 36100 Vicenza, VI Italy

**Keywords:** Parotid gland, Metastasising pleomorphic adenoma, Pleomorphic adenoma, Parotid surgery, Oncology

## Abstract

**Purpose:**

Metastasising pleomorphic adenoma (MPA) is a rare malignant tumour that can affect multiple organs with a considerable time latency compared to primary pleomorphic adenoma (PA). To date, MPA and its clinical course, including survival and relapse rates, are still poorly understood.

**Methods:**

We performed a systematic literature review following the PRISMA 2020 guidelines. The research was carried out using the PUBMED database and the following search terms: *((Metastatic) OR (metastases) OR (metastasis) OR (metastasizing)) AND (pleomorphic adenoma)*. The inclusion criteria for the final selection were: case reports or case series as study designs, availability of full text, and diseases specific to the parotid gland. Review articles, articles without full text, and studies on salivary glands other than the parotid gland were excluded.

**Results:**

A total of 908 papers were initially selected and 42 patients. The mean age at the diagnosis of MPA was 49.2 years (range 13–75 years). The average interval of onset of first metastasis was 15.7 years (range 1.4–45 years). The most common sites of metastasis were the bones (23.8%) and lungs (23.8%). In 31 patients (73.8%) a surgical treatment for complete removal of MPA was performed, while 10 patients withstood definitive or adjuvant radiotherapy. The follow up status was described only in 22 out of 40 papers where only 3 patients died from the neoplasm.

**Conclusion:**

MPA of the parotid gland is a rare condition. The transformation of a PA to MPA is unpredictable. Because of the scarcity of data in the literature, the long-term behaviour of metastatic neoplasm is uncertain. However, we found that 5 out of 19 patients experienced disease relapse after treatment. Additionally, the average survival rate for individuals with MPA of the parotid gland is 64% after 5 years. The preferred course of treatment, if possible, is the surgical removal of the neoplasm.

## Introduction

Pleomorphic adenoma (PA) is the most common benign tumor affecting salivary glands [1]. It is a mixed tumor containing both epithelial and myoepithelial elements [2] and arises in most cases in the parotid gland [3]. PA can rarely undergo malignant transformation in carcinoma ex pleomorphic adenoma (CEPA) [4] but it can also metastasize to other organs, without any malignant transformation [5]. Metastasising pleomorphic adenoma (MPA) is an uncommon malignant tumor which can affect multiple organs with considerable temporal latency compared to the primary PA [6]. In other organs, PA and MPA are histologically identical [7]. Histological signs are not reliable predictors of MPA. However, local recurrence following surgical excision has been identified as a risk factor [5]. In MPA, although the lesions are not histologically malignant, the systemic spread of the disease has a significant clinical and prognostic impact, reducing the overall survival of patients.

For most authors, total surgical resection is the preferred treatment for MPA, including total parotidectomy for primary tumors and site-specific surgery for metastases [5]. Recent evidence indicates an increasing role for adjuvant radiotherapy in reducing recurrence and relapse of the disease, although sufficient data are still lacking.

Knowledge about MPA and its clinical course remains limited, with insufficient data on survival, relapse rates, and the most suitable treatment. To address these gaps, we conducted a systematic review of the literature on parotid gland MPA. Previous studies have generally focused on MPA of the salivary glands without specifically examining MPA of the parotid gland. This review aims to investigate the distinct features of parotid gland MPA.

## Literature Review

### Materials and Method

This review followed the Preferred Reporting Items for Systematic Reviews and Meta-Analysis (PRISMA) 2020 guidelines [8]. The research was carried out using the PUBMED database with the following research string: *((Metastatic) OR (metastases) OR (metastasis) OR (metastasizing)) AND (pleomorphic adenoma)*, without any period restriction. The latest research was conducted on July 15, 2022. Article selection and phases 1 and 2 of screening were performed separately by the first two authors (S.A. and L.C.). Inclusion criteria for the first abstract reading selection were: articles in English, articles about metastatic pleomorphic adenoma, and articles about the parotid gland disease. The exclusion criteria for this phase were: articles in languages other than English, articles not concerning metastatic pleomorphic adenoma, and diseases of salivary glands other than the parotid gland. Following the initial abstract-based selection, a full-text reading (phase 2 of screening) was performed using additional criteria. The inclusion criteria for the second selection phase were: case reports or case series as study designs, full text availability and the parotid gland disease. Whereas the exclusion criteria were: reviews or other study designs, full text unavailability and diseases of salivary glands other than the parotid gland. After consolidating the selected articles, they were analysed separately by the first two authors (S.A. and L.C.) and data on the following topics were extracted: study design and year of the publication; age and gender of the patients; localization, imaging, and histologic reports of the first parotid disease and its metastasis; type of treatment and treatment’s result; potential recurrence and related treatment; as well as the follow up status. At the end of the analysis, the results from the two authors were consolidated into a single database and reviewed by a third author (S.M.).

Due to the significant heterogeneity of study populations and the predominantly qualitative nature of the collected data, no meta-analysis was planned initially or performed afterward.

## Results

**Fig. 1:**
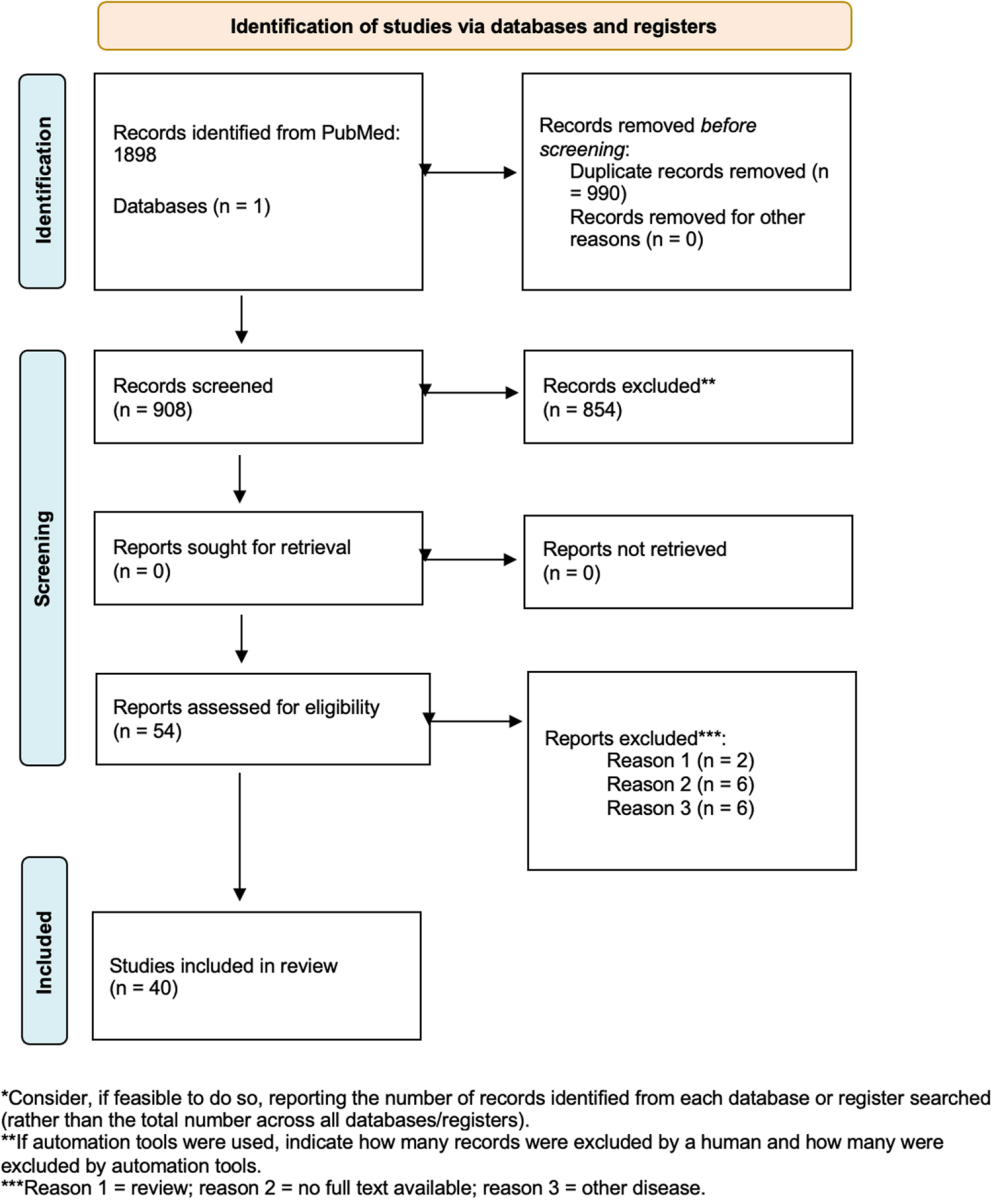
Paper selection process. *Consider, if feasible to do so, reporting the number of records identified from each database or register searched (rather than the total number cross all the database/registers). **If automation tools were used, indicate how many records were excluded by a human and how many were excluded by automation tools. ***Reason 1 = review; reason 2 = no full text available; reason 3 = other disease

Using the aforementioned research string, a total of 908 papers were chosen. First, an abstract reading selection based on the inclusion criteria was made, and 54 articles were chosen. Subsequently, a further selection was made by reading the complete articles, with the selection of 40 articles, according to the predetermined criteria. [6, 9–47]. The process of literature selection following the PRISMA statement guidelines is reported in Fig. 1.

42 patients (19 men and 23 women) were described in the selected papers. Table 1 summarizes the main features of the patients. The mean age at the diagnosis of MPS was 49.2 years (range 13–75 years). The primary parotid gland PA was in the left gland in 15 cases and in the right gland in 17 cases. In the remaining 10 cases this data was not available. Multiple surgical treatments were performed in 33.3% of the cases before the onset of MPA. The average interval of onset of first metastasis was 15.7 years (range 1.4–45 years). The site of presentation of the metastases are summarised in Table 2. The bone (23.8%) and lung (23.8%) are the most common sites of the disease.

**Table 1:** Patients’ characteristics

Author	Year	N	Sex	Age	Primary treatment of parotid disease	Histology	Time treatment-metastasis (year)	Site of first metastasis	Treat-ment	Follow up time (months)	Follow up status
Wong et al. [9]	2019	1	F	61	Total parotidectomy	PA	30	Lymph node—IFT—Bone	surgery	6	AND
Mohan et al. [10]	2018	1	M	38	Total parotidectomy	PA	21	Kidney	surgery	NA	AND
Koyama et al. [6]	2018	1	F	68	Total parotidectomy	PA	28	Bone—Kidney	–	60	AD
Nakai et al. [11]	2016	1	F	40	Enucleation	PA	12	Lung	–	3	AD
McGarry et al. [12]	2015	1	F	65	Enucleation	PA	27	Soft tissue – Muscle -Tongue—Larynx	–	3	DOD
Young et al. [13]	2015	1	F	65	Superficial parotidectomy	PA	20	Liver	–	NA	–
Mariano et al. [14]	2015	1	F	65	Enucleation	PA	3	Skin	surgery	NA	–
Abou-Foul et al. [15]	2014	1	M	57	Superficial parotidectomy	PA	7	Liver—Lung	surgery	16	AD
Tarsitano et al. [16]	2014	1	F	13	Multiple surgeries	PA	3	Nasal cavity	surgery	36	AD
Reiland et al. [17]	2012	1	M	36	Multiple surgeries	PA	21	Skin—Nasal cavity—IFT	surgery	48	AND
Zhang et al. [18]	2009	1	F	64	Multiple surgeries	PA	NA	Lung	–	NA	–
Bhutta et al. [19]	2009	1	F	75	Multiple surgeries + RT	PA	16	Skin—Kidney	surgery + RT	NA	–
Ghosh et al. [20]	2007	1	F	35	Multiple surgeries + RT	PA	6	Soft tissue	surgery + RT	NA	–
Xiao et al. [21]	2008	1	F	72	NA	PA	26	Kidney	surgery	NA	–
Steele et al. [22]	2007	1	F	43	Enucleation	PA	27	Bone—Mediastinum	surgery	NA	–
Muthusami et al. [23]	2005	1	M	31	Total parotidectomy	PA	8	Skin	surgery	24	AND
Marioni et al. [24]	2003	1	M	32	Multiple surgeries	PA	12	Nasal cavity	surgery	6	AND
Pitman et al. [25]	1991	2	F	70	Total parotidectomy	PA	1	Bone—Lung—Soft tissue—Muscle	RT	NA	–
			M	63	Multiple surgeries + RT	PA	40	Bone—Lung	–	NA	–
Girson et al. [26]	1990	1	M	33	Superficial parotidectomy	PA	16	Skin	surgery	NA	–
Ferlito et al. [27]	1991	1	F	47	Subtotal parotidectomy	PA	4	Lymph node	surgery	NA	–
Sim et al. [28]	1990	1	M	55	Multiple surgeries	PA	39	Lung	surgery	NA	–
El-Naggar et al. [29]	1988	1	F	63	Enucleation + RT	PA	9	Soft tissue	–	NA	–
Drinkard et al. [30]	1986	1	M	33	Superficial parotidectomy	PA	17	Bone	surgery	12	AND
Chen et al. [31]	1978	1	F	51	Multiple surgeries	PA	12	Bone	surgery + RT	NA	AD
Soteldo et al. [32]	2017	1	M	36	Enucleation	PA	18	Lymph node	surgery	24	AND
Chen et al. [33]	2000	1	F	51	Multiple surgeries	PA	22	Soft tissue	surgery	8	AND
Shinohara et al. [34]	1995	1	M	21	Enucleation	PA	5	Bone—Lymph node	surgery	NA	–
Sabesan et al. [35]	2007	1	M	61	Superficial parotidectomy	PA	28	Soft tissue	surgery	24	AND
Hoorweg et al. [36]	1998	2	F	61	Multiple surgeries + RT	PA	16	Lung	surgery + RT	48	AD
			M	41	Multiple surgeries + RT	PA	11	Skin	surgery	72	AND
Youngs et al. [37]	1972	1	F	28	Enucleation	PA	12	Liver	surgery	12	AND
Raja et al. [38]	2002	1	M	72	Total parotidectomy	PA	5	Lung	surgery	NA	–
Klijanienko et al. [39]	1997	1	M	56	Multiple surgeries + RT	PA	2	Soft tissue	RT	NA	DOD
Rodríguez-Fernández et al. [40]	2008	1	F	57	Superficial parotidectomy	PA	3	Lung	–	60	DOD
Schreibstein et al. [41]	1995	1	F	75	Total parotidectomy	PA	27	Bone	other treatment	14	AND
Collina et al. [42]	1989	1	M	14	Multiple surgeries	PA	6	Lymph node	surgery	36	AND
Ebbing et al. [43]	2009	1	F	49	Superficial parotidectomy	PA	29	Lung—Kidney	surgery	NA	–
Smith et al. [44]	1962	1	M	59	Multiple surgeries + RT	PA	5	Bone—Soft tissue—IFT—Orbit	surgery	NA	–
Vivian et al. [45]	2012	1	F	40	Multiple surgeries	PA	17	Kidney	other treatment	NA	–
He et al. [46]	2020	1	M	25	Multiple surgeries + RT	PA	9	Lung		NA	–
Kotani et al. [47]	2016	1	M	45	Total parotidectomy	PA	24	Brain	surgery	12	AND

PA: pleomorphic adenoma; MPA: metastasizing pleomorphic adenoma; IFT: infratemporal fossa; AND: alive without disease; AD: alive with disease; DOD: dead from disease; NA: not available

**Table 2:** Site of metastasis

Site	Cases
Bone (scapula, vertebra, mandible, maxilla, iliac, sacrum, chest)	11
Lung	11
Skin (scalp, scar, nose)	6
Lymph node	5
Kidney	5
Soft tissue (scapular region, cheek, abdomen, supraclavicular fossa)	5
Liver	3
Nasal cavity	3
Infratemporal fossa	2
Brain (middle fossa, cavernous sinus)	2
Muscle (rotator cuff muscles, iliacus)	2
Tongue	1
Larynx	1
Mediastinum	1
Orbit	1

The clinical presentation of the MPAs examined was disparate (*see *Table 3). The presence of swelling at the site of metastatic disease (parotid region, neck, scalp, abdomen) caused 19 patients to seek medical attention, while 11 patients were completely asymptomatic at the time of diagnosis. At the time of the initial clinical evaluation, some patients showed more than one symptom. Pain was found in 7 individuals, urological symptoms such as dysuria or haematuria in 6 patients, and neurological symptoms in 4 patients. Finally, 6 patients were diagnosed with MPA after the incidental finding of the lesion as a result of imaging performed for other reasons.

**Table 3:** Symptoms present at first physical examination

Symptoms	Cases
*Swelling*	19
*Pain*	7
*Urological symptoms/hematuria*	6
*Lesion detected on imaging*	6
*Neurological symptoms*	4
*No symptoms*	11

The treatment of MPA differs according to the site and stage of the disease, and the patients’ conditions. In 31 patients (73.8%) a surgical treatment for complete removal of the lesions was performed, while 10 patients underwent definitive or adjuvant radiotherapy. No information was available regarding the period between the discovery of the primary tumor and the excision surgery.

The follow up status was described only in 23 papers. This data represents a significant bias in obtaining consistent results, as it confirms the absence of solid and complete data to draw firm conclusions on the clinical history of MPA. The mean follow up period was 2.18 years (range 0.25–6 years) and it was available only for 20 patients. Six patients were reported to be alive with disease, fourteen to be alive without disease, and three to be dead from MPA. Based on the available data, only 5 patients out of 19 experienced disease relapse after treatment.

**Fig. 2:**
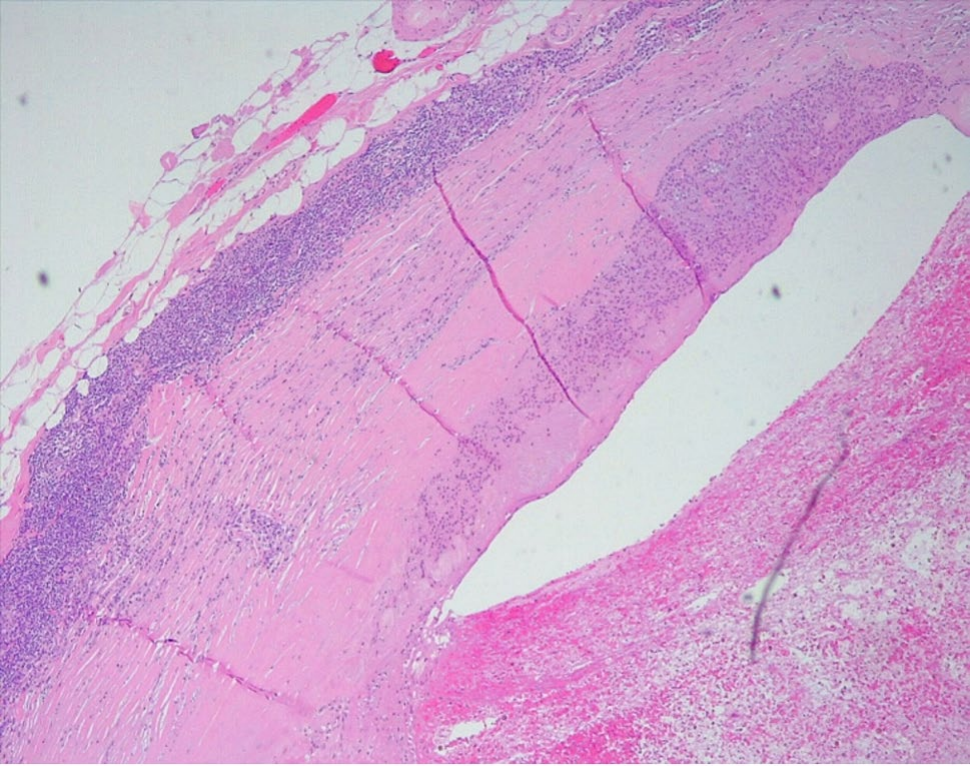
Histological features of MPA in a cervical lymph node

Figure 2 displays the Kaplan–Meier survival curve generated from the data of 20 patients. Our findings indicate that the average survival rate for individuals with MPA of the parotid gland stands at 64% after 5 years.

## Discussion

The most recent World Health Organization classification of salivary gland neoplasms categorizes an MPA as a malignant epithelial neoplasm and defines it as “a histologically benign pleomorphic adenoma that inexplicably manifests local or distant metastasis” [48].

**Fig. 3:**
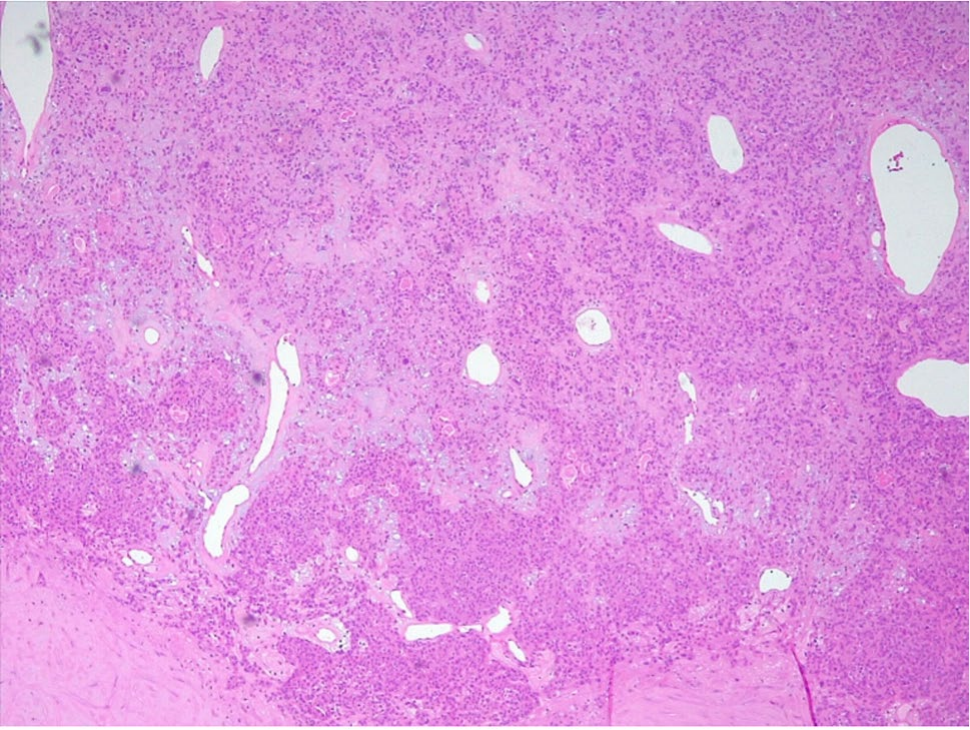
Localization of pleomorphic adenoma characterised by an epithelial and myoepithelial component without atypical features with a partly chondro-myxoid mesenchymal component

Microscopically, PA are biphasic neoplasms that can demonstrate an almost limitless number of histologic patterns. On a molecular level, PA is usually characterized by a translocation involving either PLAG1 or HMGA2, with a rearrangement identified in up to 88% of cases [7]. Current methods of histological diagnosis cannot differentiate MPA from PA (Figs. 2 and 3).

Several theories have been proposed to explain the development of metastases arising from PA:

1. MPA is a similar but unrelated tumor to conventional PA;
2. MPA represents a variant of PA with specific genetics different from the typical PLAG1/HMGA2 fusions with a greater risk of malignant behaviour;
3. MPA originates from PA after surgical manipulation which causes vascular permeation and increases the risk of metastasis;
4. All PAs have an unpredictable risk of malignant transformation that only realized in a very limited number of cases.

Several authors hypothesized that previous radiation of the primary PA or previous surgical intervention might facilitate seeding and permeation of blood or lymphatic vessels by tumor cells, followed by metastatic spread. In particular, incomplete tumor excision has been strongly related with local recurrence. Nouraei et al. [49] also noted a significant association between incomplete surgical excision of the primary lesion and development of distant metastases. The evidence available to date shows that the gold standard treatment for PA is parotid surgery, specifically the performance of superficial parotidectomy [50]. Superficial parotidectomy should be preferred for PA, because capsule rupture and spillage of the tumor are the main risk factor for recurrence of the disease [51]. These complications are more frequent when extracapsular dissection (ECD) is performed. Superficial parotidectomy demonstrates greater margins due to whole gland excision when compared to ECD, therefore reducing the risk of recurrence [52]. In case of PA, meticulous resection of the tumour with adequate margins is therefore recommended, avoiding enucleation. The analysis of surgeries performed for the treatment of primary PA shows a significant heterogeneity of treatment. Although multiple parotid surgeries are found in 16 out of 42 cases (associated with RT in 47% of cases) [16–20, 24, 25, 28, 31, 33, 36, 39, 42, 44–46], MPA also developed following single surgical procedures, either partial or total parotidectomy. Moreover, both Czader et al. [53] and Fujimura et al. [54] have described cases of MPA that developed in the absence of previous treatment of salivary neoplasia. These cases are characterized by the presence of metastatic lesions (renal and bone, respectively) that occurred before the salivary lesion was evident. Both subsequently developed rapid malignant transformation at the parotid and submandibular levels respectively, with development of Carcinoma Ex-Pleomorphic Adenoma (CEPA). These cases, although exceptional, refute the hypothesis that the development of MPA is necessarily related to surgical manipulation and support the theory that there may be an inherent tendency for malignant transformation (either as MPA or CEPA) in all PAs. Mariano et al. [14] firstly reported the results of an array comparative genomic hybridization investigation conducted on the primary parotid lesion and a skin metastasis of MPA occurred 3 years after parotidectomy. Their findings showed common genomic alterations between the two lesions and indicated a clonal origin of the secondary MPA. They also detected in the parotid tumour a specific pattern of copy number alterations (3p22.2p14.3 loss and a complex pattern of chromosome 6 deletions) that could explain the ability to metastasize.

**Fig. 4:**
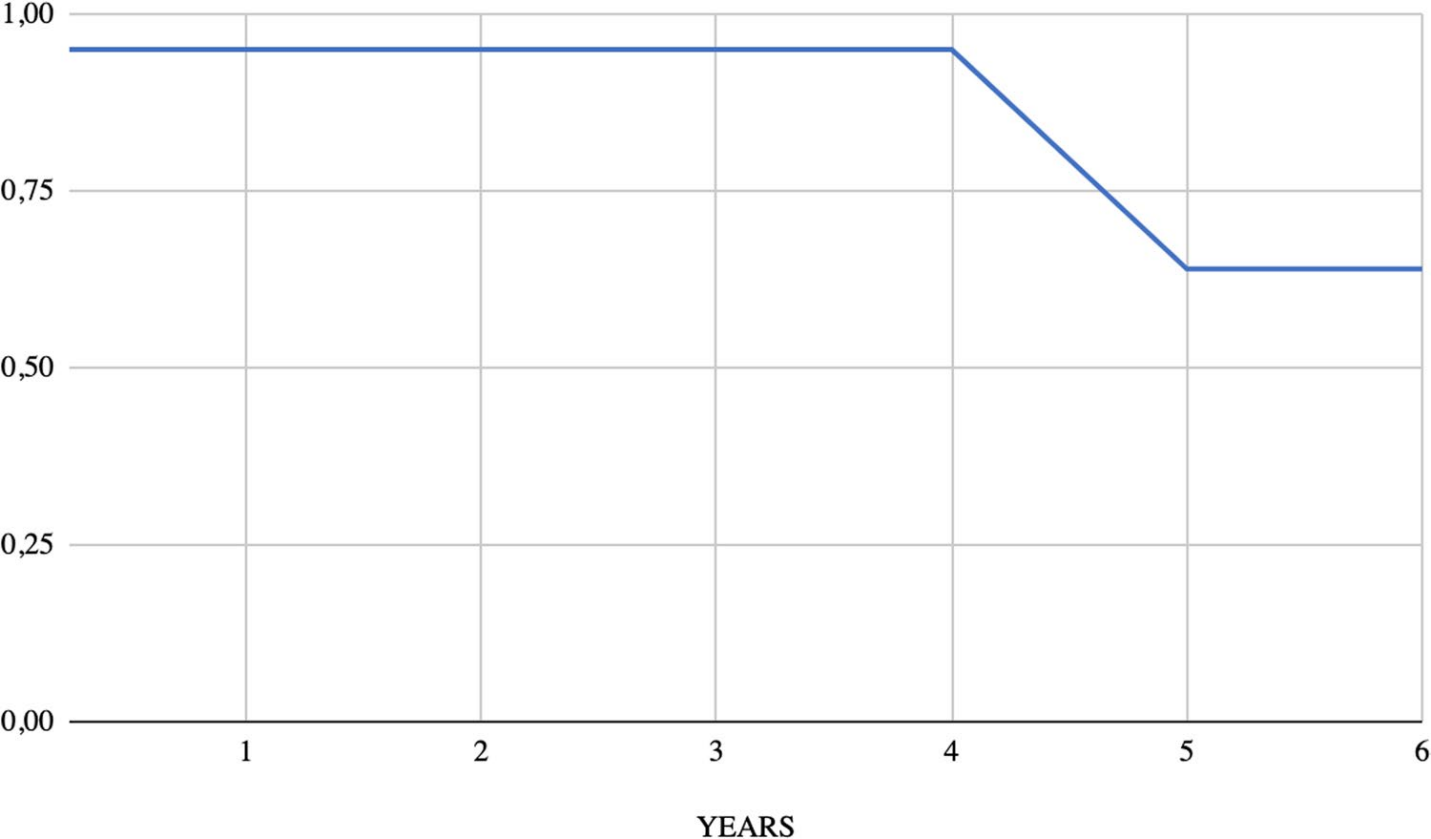
Kaplan–Meier survival curve

Further investigation of genetic elements or epigenetic influences is certainly advisable to identify possible determinants of malignant transformation (Fig. 4).

According to our data, the appearance of metastatic lesions occurred in most cases several years after treatment of parotid PA. In particular, the analysed cases show that 9 patients (21,4%) developed distant disease within 5 years [14, 16, 25, 27, 34, 38–40, 44], while in 62% of the cases metastasis occurred after more than 10 years [9–13, 17, 19, 21, 22, 24–26, 28, 30–33, 35–37, 41, 43, 45, 47]. Since the performance of systemic staging is not routine in PA patients, it is not possible to define with certainty the timing of the occurrence of asymptomatic metastases.

The most frequent sites for metastasis are bone (11 patients, 26%) and lungs (11 patients, 26%) (*see *Table 3). The distribution of metastases does not appear to be predictable; for instance, there is no correlation with the type of surgical treatment (partial, total or multiple surgeries) or time of onset (early vs late metastases). The biological mechanisms of metastasis are still unclear; the more frequent localisation to bone and lung suggests that MPA preferentially metastasises to sites prone to blood-borne metastasis, rather than MPA having a specific tropism for these organs [19]. However, the finding of skin metastases (6 patients, 14,3% of cases) [14, 17, 19, 23, 26, 36] with constant localisation near the surgical wound or the parotid region suggests the impact of surgical manipulation on the development of such lesions. In 4 out of 5 cases [14, 23, 26, 36], metastases developed at the level of the scalp, and no skin metastases were ever reported at sites outside the head and neck district.

Our data confirmed that metastases also have a slow growth, as PA in most cases. Unfortunately, data concerning follow-up are incomplete in a significant number of patients. Among those available (24 patients), only 3 patients died due to metastasis, 7 patients were alive with disease and the others 14 were alive without disease. According to data elaborated by Nouraei et al. [49] regarding the overall survival of patients with Malignant Pleomorphic Adenoma (MPA), our findings are consistent with theirs. Nouraei et al. reported a 5-year disease-specific survival rate of 58%, which is comparable to our observed rate of 64%. However, it is important to note that our study focused exclusively on MPA of the parotid gland, whereas Nouraei et al. included MPA from various locations. Furthermore, the timing of metastatic lesion presentation significantly impacts prognosis. Patients who developed metastatic lesions within 10 years of their initial primary tumor presentation had a significantly worse prognosis compared to those whose metastases were detected more than 10 years after the initial presentation of their primary PSA.

A total of 12 patients had multiple (> 1) localizations of MPA, but follow-up status was available in only 5 cases. Among the latter, two were alive without disease, two were alive with disease, and one had died due to MPA.

Regarding the treatment of metastases, 28 patients underwent surgery and 4 patients underwent surgery followed by adjuvant radiotherapy. Only 2 patients underwent definitive radiotherapy. This data supports the role of surgery as the first treatment for MPA, if possible. The choice of treatment is obviously influenced by the site and size of the lesion, as well as the general condition of the patient. Adjuvant radiotherapy treatment has been reported in a limited number of cases and there are currently no specific treatment guidelines. To date there is still insufficient evidence to strictly recommend adjuvant radiotherapy or to propose radiotherapy as a single treatment for MPA metastases.

One of the main limitations of this study is the small number of case studies available. In the current literature, a limited number of articles describe cases of MPA of the parotid gland, and the articles available are mostly case reports, with maximum two patients for each study. Moreover, these articles often do not describe the long-term follow-up of MPA patients. Finally, there are only few studies concerning the immunohistochemical and genetic profile of MPA and the available data are not sufficient to determine and predict the behaviour and the development of the metastatic disease. The absence of these data does not allow definitive conclusions to be drawn regarding the long-term behaviour of MPA, nor does it allow the effect of different treatment protocols.

Pleomorphic adenoma is the most frequent benign tumour of the salivary glands and is one of the most frequent causes of parotid surgery [55]. Considering these premises, the search for specific histological factors related to a potential malignant transformation is desirable to identify, follow up and adequately treat high-risk patients.

For the future, more studies with a larger number of patients are needed. The analysis of the currently available data does not allow certain risk factors for metastatic development to be identified. A greater understanding of the histological, immunohistochemical and genetic characteristics of these neoplasms could provide important information for predicting the transformation of PA in MPA.

Finally, a point of discussion is the follow-up management of these patients. Considering the rarity of MPA compared to the frequency of parotid PA, what should be the most prudent course of action? According to current evidence, it is not required to perform distant PET-CT in cases of PA; moreover, long-term follow-up after successful surgery for PA does not seem essential [56]. However, considering the also very large time interval between initial parotid presentation and metastasis, patients with intraoperative tumour leakage, incomplete tumour resection or local recurrence should undergo a longer follow-up with systemic imaging.

## Conclusion

MPA of the parotid gland is a very rare disease, and transformation of PA to MPA is currently an unpredictable occurrence. Metastases preferentially develop in certain sites (i.e. lung and bone) and the appearance of lesions occurs on average more than 10 years after parotid surgery. The treatment of choice for MPA is surgery; the available data do not clarify the role of definitive or adjuvant radiotherapy. Further studies with larger numbers of patients and better histologic, immunohistochemical, and genetic characterization are needed to improve our understanding of this rare condition.

## Data Availability

The data supporting the findings of this paper are available and can be provided if requested. PROSPERO does not accept scoping reviews, literature reviews or mapping reviews.
